# Hypersexuality during treatment with cariprazine in a patient with schizophrenia? A case report

**DOI:** 10.1186/s12888-023-05432-1

**Published:** 2023-12-11

**Authors:** Polona Rus Prelog, Anja Kokalj Palandacic

**Affiliations:** 1 Centre for Clinical Psychiatry, University Psychiatric Clinic Ljubljana, Ljubljana, Slovenia; 2 Medical Faculty Ljubljana, University of Ljubljana, Ljubljana, Slovenia

**Keywords:** Cariprazine, Schizophrenia, Sexual behaviour, Antipsychotic agents, Impulsive behaviour, Case report

## Abstract

**Background:**

Cariprazine is a third-generation antipsychotic with a unique mechanism of action. It functions as a partial agonist with high affinity for dopamine D2 and D3 and serotonin 5-HT1A receptors, an antagonist for 5-HT2A (moderate affinity) and 5-HT2B (high affinity) receptors. It binds to histamine H1 receptors and has a low affinity for 5-HT2C and alpha 1A-adrenergic receptors and no affinity for muscarinic (cholinergic) receptors. Among the troubling side effects, symptoms related to impulse control, such as hypersexuality, pathological gambling, compulsive shopping, compulsive eating etc., have been reported with the use of antipsychotic medications. However, no reports have been published regarding impulse control symptoms associated with cariprazine. We report a case of cariprazine-induced hypersexuality in a patient with schizophrenia, which was resolved by discontinuation of the medication.

**Case presentation:**

A 67-year-old Caucasian woman with schizophrenia was admitted to the hospital inpatient unit after she discontinued olanzapine and psychotic symptoms reappeared. Prior to that, she was in remission, taking olanzapine for approximately one year. After discontinuation, she experienced auditory hallucinations with persecutory delusions and became anergic and withdrawn, with blunted affect. Olanzapine was reintroduced, as it was proven successful in her past treatments. However, since there were no changes, especially in negative symptoms, cariprazine was added. Seven days after the introduction of cariprazine, the patient developed compulsive sexual behaviour. Therefore, cariprazine was discontinued, and the hypersexual behaviour was resolved.

**Conclusions:**

In this case report, we describe hypersexual behaviour that could potentially be induced by cariprazine. As a single case study, conclusions cannot be drawn. Controlled studies are warranted to better determine causality and the significance of this possible side-effect of cariprazine.

## Introduction

Schizophrenia is a psychiatric disorder with multiple psychiatric symptoms and presentations [1]. The disease can present with positive psychotic symptoms (i.e., hallucinations, delusions, etc.), negative symptoms (volitional impairment, such as anhedonia, blunted affect, and social withdrawal), disorganisation syndromes (formal thought disorder, disorganised behaviour, inappropriate affect) and cognitive symptoms (poor performance on tests of executive function, long-term memory, and sustained attention) [1–3]. Negative symptoms can fall into two groups: primary symptoms, which are an integral dimension of schizophrenia, and secondary symptoms, which occur as a result of positive symptoms, comorbid depression, or antipsychotic side effects [1]. It is hypothesised that negative and cognitive symptoms in schizophrenia are a result of dysfunctional dopamine 3 (D3) receptors, consequently reducing dopaminergic tone in cortical brain areas [4].

The new generation of antipsychotics with partial agonist mechanisms of action (brexpiprazole, aripiprazole, etc.), defined by some authors as “third-generation” antipsychotic (TGA) medications, have a unique mechanism of action [5]. Among them, cariprazine is a partial agonist of dopamine 2 (D2) and serotonin (5-HT)1A but it is also a partial agonist of D3 receptors, with more than a tenfold greater affinity for D3 than for D2 [6, 7]. Its receptor binding profile is similar to that of aripiprazole [7, 8]. Research data show that cariprazine is an effective treatment for patients with schizophrenia and has a positive effect on cognitive and negative symptoms of schizophrenia [8–10].

Impulse control symptoms (ICS) occurring in association with antipsychotic medication have been reported as troubling side effects and include a wide spectrum of abnormal behaviours, such as pathological gambling, hypersexuality, compulsive shopping, or eating [7, 11]. In the recent pharmacovigilance–pharmacodynamic study Fusaroli and colleagues reported, that out of 19,887 reports of impulsivity, 5,898 reports are associated with antipsychotics and 3,100 with dopamine agonists [11]. In the literature review, hypersexuality was mostly reported in patients treated with aripiprazole [12–17], while there are some cases reporting hypersexuality on risperidone [18], olanzapine [19], and clozapine [20]. However, no reports have been published regarding ICS associated with the use of cariprazine. We report a case of cariprazine-induced hypersexuality in a patient with schizophrenia, which was resolved by discontinuing the medication. Written informed consent was obtained from the patient. No ethical approval was sought since the patient understood and consented to article publication after the symptoms resolved and was without symptoms that would affect her judgment.

## Case report

A 67-year-old Caucasian woman was admitted to an acute inpatient unit of a university psychiatric hospital in February 2023 at the instigation of her son due to worsening psychosis. She has been treated for schizophrenia since she was 20 years old. She studied to be a teacher of German and Slovenian languages, worked as a bank attendant for 30 years, and retired due to chronic psychosis at 51. She had been admitted to a psychiatric hospital seven times prior due to worsening psychosis.

When she was 39 years old, she was admitted to the hospital after a suicide attempt with a gun because of visual and auditory imperative hallucinations with persecutory delusions. She was discharged with flupentixol depot 20 mg/4 weeks and clozapine 25 mg. Five years later, flupentixol depot was discontinued. She was in remission for 11 years, but after clozapine discontinuation, she developed auditory and visual hallucinations with nihilistic delusions of family members being dead. She was admitted to the hospital and discharged with aripiprazole, clozapine, and paroxetine. She was in functional remission (keeping social connections and performing household chores) until 2021 when she again discontinued medications and was admitted to the hospital due to worsening of positive symptoms. At this time, a computer tomography (CT) scan of the brain was performed that did not reveal any atrophy or other pathology. Magnetic resonance imaging (MRI) scan of the brain was not possible due to shrapnel in her body after the suicide attempt. Due to auditory hallucinations and persecutory delusions, clozapine was again introduced in combination with risperidone. Because of the negative symptoms (avolition, blunted affect, social isolation), escitalopram was introduced. She was discharged without positive or negative symptoms. She had regular appointments as an outpatient. Clozapine and risperidone were discontinued at the beginning of 2022 since she was in remission. Later, she was given olanzapine 5 mg due to insomnia. At the beginning of 2023, she abandoned her therapy with olanzapine.

At the current admission, she was admitted to the hospital with thought withdrawal and auditory hallucinations with persecutory delusions. Her mood was depressed with blunted affect. When in remission and on therapy, she was energetic and active, but after she discontinued the therapy, she became anergic, spent her days in isolation in her apartment, and became withdrawn. She was otherwise healthy with no concomitant diseases or treatments/medications. She never smoked or had any history of substance (mis)use.

**Fig. 1:**
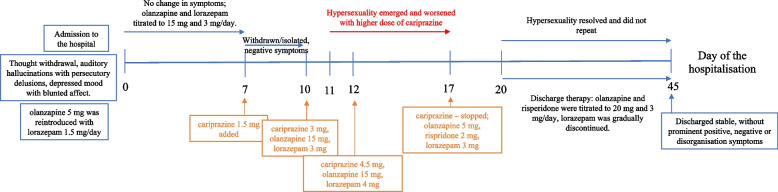
Overview timeline of patient symptoms and antipsychotic medication

During this hospitalisation, we repeated the CT scan of the brain, which was similar to that performed in 2021. When she was able to cooperate at neuropsychological testing, it revealed only cognitive symptoms with memory deficits in short and long-term recall and deficits in executive functions (planning) that were attributed to chronic schizophrenia. Blood and urine analyses were normal, and there were no signs of drug abuse. Oral olanzapine was reintroduced (Fig. 1), starting at 5 mg on the first day and increasing to 10 mg the following day, along with oral lorazepam in three daily doses, titrated up to 2 mg/day. Four days later, olanzapine was increased to 15 mg, and lorazepam was increased to 3 mg/day. Three days after olanzapine was raised, she was still withdrawn and isolated. Hence, 1.5 mg of cariprazine was introduced. After three days, the dose was increased to 3 mg. Four days after the cariprazine introduction, she was found kissing and stroking another female patient in the ward. Hypersexuality was first considered a part of the disorganisation syndrome of schizophrenia; hence, cariprazine was raised to 4.5 mg. Afterwards, the patient was found masturbating naked in her bed and in the middle of the ward on several occasions. During her stay in the next few days/weeks, she was found naked in her room several times or even came out naked. Even reminded of the inappropriateness, she ignored the reminders and repeated the actions (stroking other patients and even medical stuff), taking her clothes off, walking around the ward naked, and compulsively masturbating.

Since the symptoms did not resolve with cariprazine and olanzapine, we cross-tapered olanzapine for risperidone, but the behaviour continued; consequently, after three days, cariprazine was paused and ended. The hypersexual behaviour stopped/resolved within 72 h. However, some delusions (and possibly auditory hallucinations that she did not confirm) persisted. Olanzapine was raised to 20 mg, however, due to persistent delusions, risperidone was titrated up to 3 mg. Psychosis gradually resolved while the hypersexual behaviour was never repeated. Since the symptoms resolved, no additional tests were warranted after cariprazine withdrawal. The patient was discharged in April 2023, stable, without prominent positive, negative, or disorganisation symptoms, and received olanzapine 20 mg and risperidone 3 mg. Since then she has had regular outpatient follow-ups and is in stable remission with the same treatment. After reviewing the patient’s medical history for past episodes of hypersexuality, we noticed the recurrence of depressed mood with nihilistic delusions and psychomotor retardation, and in addition to antipsychotic therapy, antidepressants were prescribed. Hypersexual behaviour or other ICSs were not described.

## Discussion

Sexual function is thought to be mediated by dopamine and serotonin [21]. Sexual arousal is regulated by dopamine through the mesolimbic-mesocortical system, and antipsychotics (especially first-generation antipsychotics) can reduce sexual drive by D2 blockade [11, 19]. However, partial agonism of second-generation antipsychotics (SGA) and TGA on dopamine receptors, particularly on D3, could induce ICS, since D3 receptors are predominantly found in cerebral circuits involved in the reward system [7, 11, 19]. Sexual function is also promoted by 5-HT1A agonism and 5-HT2A antagonism [22, 23]. Drugs, such as aripiprazole and possibly cariprazine, with their partial agonism on 5-HT1A and D3 and antagonism on 5-HT2A, could cause ICS [6, 7, 13, 14].

Hypersexuality is a well-documented symptom among ICS. It can be a symptom of schizophrenia or a side effect of TGA [21]. ICS frequently appears in Parkinson’s disease when patients are treated with dopamine agonists [11, 24]. The literature suggests that higher agonist affinity for D3 receptors results in higher rates of ICS, especially in patients with Parkinson’s disease [7, 11]. Furthermore, a recent pharmacovigilance-pharmacodynamic study performed by Fusarolli and colleagues suggested that the agonism of 5-HT1A could be the second mechanism associated with ICS [11]. The hypothesis is explained based on brexpiprazole and lurasidone dopaminergic profile, which is different from aripiprazole and cariprazine, but they do share the agonism on 5-HT1A, which could inhibit the pathway responsible for impulse control [11].

Considering the similarity of the pharmacological profile of cariprazine and aripiprazole and the number of reported ICS associated with dopamine agonists, it is surprising that no case reports have yet been published regarding hypersexuality or other ICS associated with cariprazine [11]. However, Zazu and colleagues reviewed the European pharmacovigilance database and found seven cases of cariprazine-related ICS, mostly in patients with schizoaffective disorder [7]. The reported ICS were hypersexuality, compulsive shopping, and binge eating [7].

This case presents a patient with schizophrenia who developed hypersexual behaviour shortly after initiating treatment with cariprazine. We first introduced olanzapine, which was efficient in the past. Olanzapine has a strong antidopaminergic and antiserotonergic profile, and as proposed by Herguner, the blockade of serotonergic neurotransmission could increase dopamine in the mesocortical pathway and cause ICS [14, 19]. Olanzapine worked well on positive symptoms of schizophrenia, and no side effects, i.e., extrapyramidal signs, akathisia, hypersexuality, or other ICS, were reported by the patient or her relatives. For anxiety relief lorazepam was used in combination with olanzapine. The concomitant use of any form of olanzapine and lorazepam is not recommended by some authors, due to fatal cases reported after intramuscular application of olanzapine and lorazepam [25]. In our case, the oral form of both medications was introduced in the hospital, where the patient was regularly observed by the nurses and doctors, to avoid potential sedation or other adverse events. Because negative symptoms were still present (such as blunted affect, alogia, avolition, and social isolation), we introduced cariprazine since it is supposed to have a positive effect on the negative and cognitive symptoms of schizophrenia [8–10, 26]. After cariprazine was introduced, symptoms of hypersexuality emerged, hence we decided to discontinue cariprazine. The patient was discharged on olanzapine and risperidone. Polypharmacy had to be used since the Slovenian Mental Health Act strictly defines indications for exceeding the maximum doses of antipsychotics. The maximum dose can be exceeded when no other effective treatment method is available, when it is necessary for effective treatment, and if the patient has given written consent. When all three criteria are met, a committee of at least three independent doctors has to be consulted and give their approval for exceeding the maximum dose [27, 28]. Olanzapine was a first-choice antipsychotic for our patient since it was effective in the past. However, despite the maximum dose of olanzapine and a small dose of risperidone, some psychotic symptoms persisted. The criteria for exceeding the maximum dose were not met, since the patient strictly declined clozapine; to achieve better compliance, we raised the dose of risperidone. Even though polypharmacy can sometimes represent a potential threat for additive adverse effects of antipsychotics, it is sometimes unavoidable because of our legislation [28].

The patient was diagnosed with schizophrenia when she was 20 years old. She had auditory hallucinations, delusions, and diminished emotional expression, with a decline in functioning (with social withdrawal, and she was unable to do household chores). However, after reviewing the patient's medical history, in this hospitalisation, a new symptom of hypersexuality emerged. Hypersexuality could be a part of mania; hence, we should consider bipolar or schizoaffective disorder as a differential diagnosis. According to the Diagnostic and Statistical Manual of Mental Disorders, Fifth Edition (DSM-5), at least one symptom of schizophrenia (delusions, hallucinations, disorganised speech, grossly disorganised behaviour/catatonia) should be present for at least one month. However, according to the International Classification of Diseases 11th Revision (ICD 11), at least two symptoms of schizophrenia should be present for one month (one should be out of delusions, hallucinations, disorganised speech, the experience of influence/passivity/control) [29, 30]. In the past, the patient had nihilistic delusions with anhedonia and a depressed mood concurrent with the DSM-5 criterion A of schizophrenia. Symptoms of mood disorder were prominent only during the active phase of positive symptoms, hence criterion C (the symptoms of major mood disorder are present for the majority of the total duration of the active and residual portions of the illness) for schizoaffective disorder in DSM-5 is not met. Psychotic symptoms were more prominent and persisted even after the resolution of the symptoms of major mood disorder. Furthermore, the presence of positive, negative, and cognitive symptoms, had an impact on lower functioning at work, self-care, and interpersonal relations [29, 31]. The diagnosis of schizoaffective disorder and uni-/bipolar mood disorder was ruled out.

A behavioural variant of frontotemporal dementia (bvFTD) should also be considered. Hypersexuality and disinhibition without evident cognitive decline are also characteristics of bvFTD [32, 33]. An MRI and (18F)-fluorodeoxyglucose positron emission tomography (FDG-PET) studies have characterised patterns of neurodegeneration and hypometabolism that define the different clinical and pathological variants of FTD [34]. The patient had a contraindication for an MRI, hence an FDG-PET should be performed to exclude bvFTD. Unfortunately in Slovenia, the FDG-PET is rarely available. Based on the resolution of the hypersexuality after cariprazine discontinuation, the CT scan without relevant atrophy, and psychological testing with a mild cognitive decline typical for chronic schizophrenia, a diagnosis of bvFTD seemed unlikely. Furthermore, the symptoms never returned after cariprazine discontinuation. If in the future the patient presents with a similar clinical picture, the FDG-PET is to be done.

Since cariprazine has a similar receptor profile as aripiprazole, drug-induced dopamine hypersensitivity in schizophrenic patients should also be considered as a part of the differential diagnosis [35]. A worsening of psychosis after adding or switching from another antipsychotic agent to aripiprazole was previously described [36, 37].

Considering the published case of ICS caused by aripiprazole and the time in which the ICS resolved [17], we looked at the published data on cariprazine metabolism. Cariprazine has a half-life of 2–4 days, and its metabolites desmethylcariprazine (DCAR) and didesmethylcariprazine (DDCAR) have half-lives of 1–2 days and 1–3 weeks, respectively [38]. DCAR is a primary metabolite of cariprazine formed by demethylation, while DDCAR is formed from DCAR by a further demethylation process [39]. It took 72 h for symptoms to completely resolve. Considering the half-life of cariprazine regarding ICS dismissal in our patient, we could speculate that in three days, the cariprazine concentration fell below 3 mg, which was the concentration when the first symptoms of hypersexuality appeared.

There are also limitations to be mentioned; the first limitation of this article is that it is based on the description of one patient, and it is hard to generalise the presentation to the whole patient population. The second limitation is that cariprazine was taken together with olanzapine. Consequently, we cannot rule out whether the olanzapine-cariprazine interaction contributed to hypersexuality. Third, since the ICS disappeared after the discontinuation of cariprazine and the behaviour gave the patient feelings of shame and presented significant stress, a rechallenge test was unethical. Regarding all the limitations and plausibility of cariprazine directly causing hypersexuality, we used the Naranjo Algorithm or Adverse Drug Reaction Probability Scale, where the score showed a possible causality (4 points) [40].

## Conclusion

In conclusion, to the best of our knowledge, we report the first case of hypersexuality that may occur because of treatment with cariprazine. The current evidence is limited, and causality cannot be concluded from a case report. More studies on larger populations are required to investigate the possible associations of antipsychotic medications, including cariprazine, with ICS. In addition, measuring levels of cariprazine and its metabolites in the serum could make the correlation between cariprazine and ICS more concise. Further research is warranted to define possible correlates and neurobiological evidence for hypersexual behaviour in patients treated with cariprazine.

## Data Availability

The datasets used and/or analysed during the current study are available from the corresponding author upon reasonable request.

## Abbreviations

D3: Dopamine 3 receptors
TGA: Third-generation antipsychotic medication
D2: Dopamine 2 receptor
5-HT: Serotonin or 5-hydroxytryptamine
ICS: Impulse control symptoms
CT: Computer tomography
MRI: Magnetic resonance imaging
SGA: Second-generation antipsychotics
SSRI: Serotonin reuptake inhibitors
DSM-5: The Diagnostic and Statistical Manual of Mental Disorders, Fifth Edition
ICD 11: International Classification of Diseases 11th Revision
bvFTD: Behavioural variant of frontotemporal dementia
FDG-PET: (18F)-fluorodeoxyglucose positron emission tomography
DCAR: Desmethylcariprazine
DDCAR: Didesmethylcariprazine

## References

[1] Demyttenaere K, Anthonis E, Acsai K, Correll CU. Depressive symptoms and PANSS symptom dimensions in patients with predominant negative symptom schizophrenia: a network analysis. Front Psychiatry. 2022;13:795866. 10.3389/fpsyt.2022.795866. 10.3389/fpsyt.2022.795866 PMC9081724 35546936

[2] Mosolov SN, Yaltonskaya PA. Primary and secondary negative symptoms in schizophrenia. Front Psychiatry. 2022;12:766692. 10.3389/fpsyt.2021.766692. 10.3389/fpsyt.2021.766692 PMC8761803 35046851

[3] Jauhar S, Johnstone M, McKenna PJ (2022). Schizophrenia Lancet.

[4] Kehr J, Wang FH, Ichinose F, Yoshitake S, Farkas B, Kiss B, Adham N (2022). Preferential Effects of Cariprazine on Counteracting the Disruption of Social Interaction and Decrease in Extracellular Dopamine Levels Induced by the Dopamine D3 Receptor Agonist, PD-128907 in Rats: Implications for the Treatment of Negative and Depressive Symptoms of Psychiatric Disorders. Front Psych.

[5] Rancans E, Dombi ZB, Barabássy Á (2022). Dosing cariprazine within and beyond clinical trials: recommendations for the treatment of schizophrenia. Front Psych.

[6] Schölin JS, Cruz JR, Hjorth S. Successful switching from risperidone to cariprazine in a schizophrenic patient with pronounced functional deficit. Case report. Front Psychiatry. 2023;14:1155395. 10.3389/fpsyt.2023.1155395. 10.3389/fpsyt.2023.1155395 PMC10067887 37020736

[7] Zazu L, Morera-Herreras T, Garcia M, Aguirre C, Lertxundi U (2021). Do cariprazine and brexpiprazole cause impulse control symptoms? A case/noncase study. Eur Neuropsychopharmacol.

[8] Corponi F, Fabbri C, Bitter I, Montgomery S, Vieta E, Kasper S, Pallanti S, Serretti A (2019). Novel antipsychotics specificity profile: a clinically oriented review of lurasidone, brexpiprazole, cariprazine and lumateperone. Eur Neuropsychopharmacol.

[9] Csehi R, Dombi ZB, Sebe B, Molnár MJ. Real-life clinical experience with cariprazine: a systematic review of case studies. Front Psychiatry. 2022;13:827744. 10.3389/fpsyt.2022.827744. 10.3389/fpsyt.2022.827744 PMC8970284 35370825

[10] André V, Vannucchi T, Taddeucci C, Tatini L (2022). Functional and symptomatic improvement with cariprazine in various psychiatric patients: a case series. Front Psych.

[11] Fusaroli M, Giunchi V, Battini V, Gringeri M, Rimondini R, Menchetti M, Radice S, Pozzi M, Nobile M, Clementi E, De Ponti F (2023). Exploring the underlying mechanisms of drug-induced impulse control disorders: a pharmacovigilance-pharmacodynamic study. Psychiatry Clin Neurosci.

[12] Bulbena-Cabré A, Bulbena A. Aripiprazole-Induced Hypersexuality. Prim Care Companion CNS Disord. 2016;18(6). 10.4088/PCC.16l01983. 10.4088/PCC.16l01983 28033456

[13] Kozian R (2020). Sexuelle Enthemmung bei Therapie mit Aripiprazol [Hypersexuality Induced by Aripiprazole]. Psychiatr Prax..

[14] Das S, Chatterjee SS, Bagewadi V (2017). Aripiprazole induced hypersexuality, when we should be cautious?. Asian J Psychiatr.

[15] Dhillon R, Bastiampillai T, Cao CZ, Eckert TG, Tibrewal P (2017). Aripiprazole and impulse-control disorders: a recent FDA warning and a case report. Prim Care Companion CNS Disord.

[16] Reddy B, Ali M, Guruprasad S, Das S (2017). Hypersexuality induced by Aripiprazole: two case reports and review of the literature. Asian J Psychiatr.

[17] Priya L, Moorthy B (2021). A case of hypersexuality in a patient receiving aripiprazole for schizophrenia. Case Rep Psychiatry.

[18] Davidson CK, Johnson T, Jansen K (2013). Risperidone-induced hypersexuality. Br J Psychiatry.

[19] Hergüner S (2010). Excessive masturbation associated with olanzapine in a pediatric case. Prog Neuropsychopharmacol Biol Psychiatry.

[20] Thomson SR, Patil N, Ommurugan B, Bhandari RK (2018). A case of hyper sexuality probably associated with clozapine. Psychopharmacol Bull.

[21] Stefanou MI, Vittore D, Wolz I, Klingberg S, Wildgruber D (2020). Recurrent episodes of paraphilic behavior possibly associated with olanzapine and aripiprazole treatment in a patient with schizophrenia. Front Psychiatry.

[22] Moses TEH, Javanbakht A (2023). Resolution of Selective Serotonin Reuptake Inhibitor-Associated Sexual Dysfunction After Switching From Fluvoxamine to Fluoxetine. J Clin Psychopharmacol.

[23] Meston CM, Frohlich PF (2000). The neurobiology of sexual function. Arch Gen Psychiatry.

[24] Grall-Bronnec M, Victorri-Vigneau C, Donnio Y, Leboucher J, Rousselet M, Thiabaud E, Zreika N, Derkinderen P, Challet-Bouju G (2018). Dopamine agonists and impulse control disorders: a complex association. Drug Saf.

[25] Sadock BJ, Sadock VA. Kaplan and Sadock's pocket handbook of clinical psychiatry. Philadelphia: Lippincott Williams & Wilkins; 2018.

[26] Németh G, Laszlovszky I, Czobor P, Szalai E, Szatmári B, Harsányi J, Barabássy Á, Debelle M, Durgam S, Bitter I, Marder S, Fleischhacker WW (2017). Cariprazine versus risperidone monotherapy for treatment of predominant negative symptoms in patients with schizophrenia: a randomised, double-blind, controlled trial. Lancet.

[27] (Mental Health Act) Zakon o duševnem zdravju. Uradni list RS st. 77/08: http://www.pisrs.si/Pis.web/pregledPredpisa?id=ZAKO2157#

[28] Kokalj A, Rijavec N, Tavčar R. Delirium with anticholinergic symptoms after a combination of paliperidone and olanzapine pamoate in a patient known to smoke cannabis: an unfortunate coincidence. BMJ Case Rep. 2016; 22: 10.1136/bcr-2016-214806. 10.1136/bcr-2016-214806 PMC4932405 27335358

[29] American Psychiatric Association (2022). Diagnostic and statistical manual of mental disorders (5th ed., text rev.).

[30] International Classification of Diseases, Eleventh Revision (ICD-11), World Health Organisation (WHO) 2019/2021. https://icd.who.int/browse11.

[31] Malaspina D, Owen MJ, Heckers S, Tandon R, Bustillo J, Schultz S, Barch DM, Gaebel W, Gur RE, Tsuang M, Van Os J, Carpenter W (2013). Schizoaffective Disorder in the DSM-5. Schizophr Res.

[32] Dubljević V (2020). The principle of autonomy and behavioural variant frontotemporal dementia. J Bioeth Inq.

[33] Jazi AN, Shebak SS, Kim KY. Treatment of Hypersexuality in an Elderly Patient With Frontotemporal Dementia in a Long-Term Care Setting. Prim Care Companion CNS Disord. 2017;19(3). 10.4088/PCC.16l02031. 10.4088/PCC.16l02031 28564516

[34] Whitwell JL (2019). FTD spectrum: Neuroimaging across the FTD spectrum. Prog Mol Biol Transl Sci.

[35] Chouinard G, Samaha AN, Chouinard VA, Peretti CS, Kanahara N, Takase M, Iyo M (2017). Antipsychotic-induced dopamine supersensitivity psychosis: pharmacology, criteria, and therapy. Psychother Psychosom.

[36] Adan-Manes J, Garcia-Parajua P (2009). Aripiprazole in combination with other antipsychotic drugs may worsen psychosis. J Clin Pharm Ther.

[37] Takase M, Kanahara N, Oda Y, Kimura H, Watanabe H, Iyo M (2015). Dopamine supersensitivity psychosis and dopamine partial agonist: a retrospective survey of failure of switching to aripiprazole in schizophrenia. J Psychopharmacol.

[38] Andrade C (2022). Practical psychopharmacology: using a knowledge of pharmacokinetics to more rapidly stabilize patients at lower drug doses. J Clin Psychiatry.

[39] Kiss B, Némethy Z, Fazekas K, Kurkó D, Gyertyán I, Sághy K, Laszlovszky I, Farkas B, Kirschner N, Bolf-Terjéki E, Balázs O (2019). Preclinical pharmacodynamic and pharmacokinetic characterization of the major metabolites of cariprazine. Drug Des Dev Ther.

[40] Naranjo CA (1981). A method for estimating the probability of adverse drug reactions. Clin Pharmacol Ther.

